# Perceived competences, attitudes, and training needs in conflict management among a cohort of Italian physiotherapists: A cross-sectional survey study

**DOI:** 10.1371/journal.pone.0306095

**Published:** 2024-07-26

**Authors:** Simone Battista, Annalisa De Lucia, Marco Testa, Valeria Donisi

**Affiliations:** 1 School of Health and Society, Centre for Human Movement and Rehabilitation, University of Salford, Salford, Greater Manchester, United Kingdom; 2 Section of Clinical Psychology, Department of Neurosciences, Biomedicine and Movement Sciences, University of Verona, Verona, Italy; 3 University of Genova, Department of Neuroscience, Rehabilitation, Ophthalmology, Genetics, Maternal and Child Health, Genova, Italy; Iran University of Medical Sciences, ISLAMIC REPUBLIC OF IRAN

## Abstract

Conflict management is rarely explored among physiotherapists though they often work in teams. Hence, this study explored attitudes, perceived competencies, beliefs, training experiences, and needs in conflict management among Italian physiotherapists. We conducted a cross-sectional online survey study between June and September 2023 among Italian physiotherapists. The survey instrument comprised four sections. Section 1: Socio-Demographic and Professional Data: Explored participant profiles and conflict frequency. Section 2: Attitudes and Competences: assess conflict-related behaviours and management styles (Likert Scale). Section 3: Training Experiences and Needs: Evaluated training importance and conflict-related issues with other professionals (Likert Scale). Section 4: Beliefs About Factors: Participants rated (0–10) factors influencing conflict management and its impact on care and well-being. Descriptive analyses were performed, presenting continuous data as mean (SD) and categorical data as frequencies/percentages. Likert scale responses were dichotomised (agreement/disagreement), and consensus was defined as ≥70% agreement. Median, quartiles, and box-and-whisker plots depicted responses were used for 0-to-10 scales. Physiotherapists (n = 203; mean age: 39±10.40) generally leaned towards a constructive communication style, characterised by compromise and collaboration, viewing conflict management as an opportunity to grow. There was a disparity between their exhibited behaviours and self-assessment of appropriateness in conflict resolution. Only 27.6% considered their conflict resolution skills as satisfactory. However, 85.7% acknowledged the significance of being trained in conflict management. Challenges were evident in conflicts within interprofessional relationships and communication with superiors. Both personal and organisational factors were identified as influencing conflict management, with participants recognising the detrimental impact of conflicts on their well-being and patient care. This study highlighted educational gaps in conflict management among Italian physiotherapists, showing areas of improvement in their training. Our results suggested that physiotherapists might need additional training in conflict management to enhance workplace well-being and the quality of care provided.

## Introduction

Healthcare systems are intricate entities characterised by numerous dynamic interactions among diverse components, including healthcare providers, consumers, organisations within the healthcare system, and contextual factors [1]. However, interpersonal conflicts often arise in the healthcare setting [2, 3]. Interpersonal conflicts are generally defined as a felt struggle between interdependent individuals due to perceived incompatible differences in goals, values, and beliefs, scarce resources, and interference from others in achieving goals [4]. These conflicts may occur between healthcare professionals and other staff members, healthcare teams, but even between healthcare professionals and patients (or their caregivers). The consequences of conflicts extend beyond emotional and physical tolls, impacting healthcare providers’ well-being, job satisfaction, and ability to work [5]. Moreover, conflicts in clinical settings pose a substantial threat to patients’ well-being [6].

Given the potential negative impact of conflict on the quality of healthcare, recent attention has been given to considering preventive actions and training programs for the management of conflicts among healthcare professionals [7, 8]. However, the area of conflict management is an emerging and still poorly explored field that needs further development, especially in some healthcare context, such as in the rehabilitation field. Specifically, interprofessional rehabilitation teams consist of many professional groups, including nurses, doctors, and allied health professionals such as physiotherapists working together to answer patients’ needs [9].

Hence, the general recent interest in interprofessional collaboration and its effective and growing role in managing long-term conditions encouraged physiotherapists to increase their interactions with other healthcare professionals [10]. However, only a few papers have specifically investigated physiotherapists’ perceptions of interprofessional and interprofessional working in clinical settings, exploring the skills and factors considered relevant to functional working collaboration [11–13]. However, these studies have only marginally considered attitudes, beliefs, and needs towards the specific topic of conflict management.

Considering this background, the current study aimed to investigate: (i) attitudes towards interpersonal conflicts and perceived competences in conflict management; (ii) beliefs about interpersonal conflicts; and (iii) training experiences and needs for conflict management among a cohort of Italian physiotherapists.

## Material and methods

### Research design

We developed a web-based cross-sectional survey study directed at Italian physiotherapists following the recommendation of the ‘International Handbook of Survey Methodology’ [14]. The study received approval from the University Research Ethics Committee (data of approval: 22/05/2023 code: XXX2023.32) at the University of XXX (XXX, XXX). Participants received and download the electronic informed consent sheet in the first page of the survey. Then, they decided whether to give electronic informed consent before participation by answering Yes/No to the sentence “I consent to participate in this study after having read and understood the information contained in the informed consent form, and I declare that I have viewed the information for the processing of personal data contained therein.” The study was conducted in accordance with the Declaration of Helsinki and reported following the Strengthening the Reporting of Observational Studies in Epidemiology (STROBE) recommendation [15].

### Survey instrument creation

The survey instrument was devised by two authors with different expertise, SB and VD. SB is a physiotherapist with a PhD in ‘Neurosciences’ and ‘Medical Science’, specialised in the ‘Rehabilitation of Rheumatic and Musculoskeletal Diseases’. VD is a clinical psychologist holding a PhD in ‘Psychiatric and Psychological Science’ with expertise in communication training for healthcare professionals, including group dynamics and conflict resolution topics. Apart from initial questions regarding socio-demographic and professional data to describe the characteristics of the participants, different topics have been explored in the survey: (i) attitudes toward conflict competencies in conflict management, (ii) training experiences and needs toward conflict management, (iii) belief on conflict in the healthcare sectors. The questions and items included in the survey instrument were crafted with input from the literature on conflict management in healthcare [5, 16–19]. The draft of the survey instrument was subsequently pilot tested on five physiotherapists for face validity. Each physiotherapist received the survey instrument draft and was asked to complete it independently, providing feedback to the researchers about content validity and comprehensibility. Their feedback was integrated in the and the final survey was composed of four sections, explained hereafter. The translation of the survey instrument into English has been reported in S1 File. The survey instrument was delivered through ‘Microsoft Form 365,’ a secure and safe web-based application for online survey instruments, adhering to European GDPR and not allowing any missing data as data are not saved until the participants have compiled them in all sections.

#### Section 1: Socio-demographic, professional data and conflict frequency

In this section, participants were asked about their professional profile, type of work, age, gender, years of professional experience, the other professional figures they work with most frequently, and the frequency of conflict situations they are involved in.

#### Section 2: Attitudes towards and perceived competences in conflict management

This section required participants to assess their attitudes toward conflicts and perceived competencies in conflict management utilising a 5-point Likert scale ranging from ‘completely disagree’ to ‘completely agree.’ First, participants were assessed as to what extent they believed they exhibited certain conflict-related behaviours. The list of behaviours included basic communication skills such as listening to the interlocutor, emotional competences being aware of one’s emotions in relationships, and specific conflict management skills supporting and valuing one’s ideas while respecting others’, collaborating with other healthcare professionals, negotiating, giving positive feedback, being able to receive feedback, finding a compromise regarding a different opinion with a colleague, and managing a conflict situation constructively. Participants were also asked to indicate which of the previous behaviours they believed would be more appropriate for conflict management.

Additionally, participants identified the conflict management styles that best represented their actions, including the tendency to assert themselves in an authoritative way, to conform to others’ opinions, to avoid conflict, to find a compromise between their and someone else’s opinion, to seek another person’s opinion to reach a consensus, to seek collaboration and exchange with other people. These styles were introduced in the survey according to the Thomas-Kilmann Conflict Management Model [19, 20], which distinguishes different interpersonal conflict-handling modes: competing, collaborating, compromising, avoiding, and accommodating. Moreover, the last item focussed on the tendency to engage because “I see [the conflict] as an opportunity for growth. This item has been added because a proactive and constructive attitude towards conflict, conceived as an inevitable and not necessarily adverse component of interpersonal relationships, is suggested to result in better outcomes than management styles based on avoidance or prevarication [21].

#### Section 3: Training experiences and needs in conflict management

In this section, participants were asked to rate their perceived level of skills in dealing with conflict and the importance for their profession of being trained in conflict management with a 5-point Likert scale ranging from ‘completely disagree’ to ‘completely agree.’ Moreover, participants were also asked potential specific training needs indicating to what extent they found it difficult to deal with conflicts with colleagues of the same profession, colleagues from other professions, supervising colleagues, and colleagues supervised by the participant.

#### Section 4: Beliefs about factors influencing conflict management

Participants were asked to rate to what extent they believed factors might influence conflict management in the workgroup. Factors included time spent in professional discussions, perceived workload, personal relational skills, patient complexity, organisational variables, leadership, and resource scarcity. The second question pertained to the impact of conflict management on the care provided, the use of a biopsychosocial approach, and the participant’s well-being. Each factor and impact area was evaluated on a scale from 1 to 10.

### Population and recruitment methods

The survey was distributed via social media (specifically Facebook groups and healthcare professional Telegram groups) and shared among colleagues through word of mouth between June 1 and September 30, 2023. Participation in the survey required individuals to meet the following inclusion criteria: 1) being registered Italian physiotherapists, 2) working in a team (either full-time or part-time), and 3) consenting to study participation and data collection by providing electronic informed consent. These criteria were verified at the beginning of the survey, prior to section 1, by asking participants about their registration status as physiotherapist, if they work in team, and electronic consent to participate. Exclusion criteria included: 1) not being a registered Italian physiotherapist, 2) not working in a team, and 3) not providing consent for study participation and data collection. Participants who did not meet the inclusion criteria were directed to a ’Thank-you’ page and were unable to proceed further.

### Data analysis

Data were extracted into an Excel spreadsheet, and descriptive analyses were conducted. Continuous data were reported as mean and standard deviation (SD). Categorical data were reported as frequencies and percentages. Likert scale responses were tabulated, and percentages for each response were computed. Responses were categorised into completely positive (completely agree and agree) and completely negative (completely disagree and disagree and neutral), and percentages were calculated based on the sum. These percentages were then represented on a dichotomous bar histogram. The overall consensus with each statement was investigated. In the absence of a standard threshold, we defined a ≥ 70% agreement with a statement as consensus [22, 23]. For responses on a scale of 1 to 10, the median, first, and third quartiles were calculated for each item, and the data were represented with a box-and-whisker plot.

## Results

### Section 1: Socio-demographic and professional characteristics of the sample

We collected data from 203 Italian physiotherapists (mean age: 39 years, SD ±10.40; 60.09% female, 35.96% male, and 3.95% prefer not to declare). Table 1 reports the socio-demographic and professional characteristics of the sample.

**Table 1:** Descriptive statistics.

Characteristics	N = 203
**Work Setting, N(%)**	
Always work in teams or workgroups	108 (53.3)
Work in teams or groups sometimes	95 (47.7)
**Age (mean ± SD)**	38.7 ± 10.33
**Gender Identification, N(%)**	
Female	122 (60.09)
Male	73 (35.96)
Prefer not to declare	8 (3.95)
**Work Experience, N(%)**	
More than 10 years	106 (52.2)
Between 6 and 10 years	54 (26.6)
Between 1 and five years	35 (17.2)
Less than 1 year	8 (4.0)
**Collaboration with Professional Figures, N(%)**	
Other physiotherapists	158 (77.8)
Physicians	180 (88.7)
Nurses	142 (70.0)
Healthcare assistants	198 (97.5)
Psychologists	52 (25.6)
Professional educators	32 (15.8)
Occupational therapists	58 (28.6)
Administrative staff	1 (1)
**Frequency of Conflict in Team, N(%)**	-
Never	5 (2.5)
Rarely	58 (28.6)
Sometimes	65 (32.0)
Often	58 (28.6)
Daily	17 (8.4)

N, number; SD, standard deviation

### Section 2: Attitudes toward and perceived competences in conflict management

**Fig 1:**
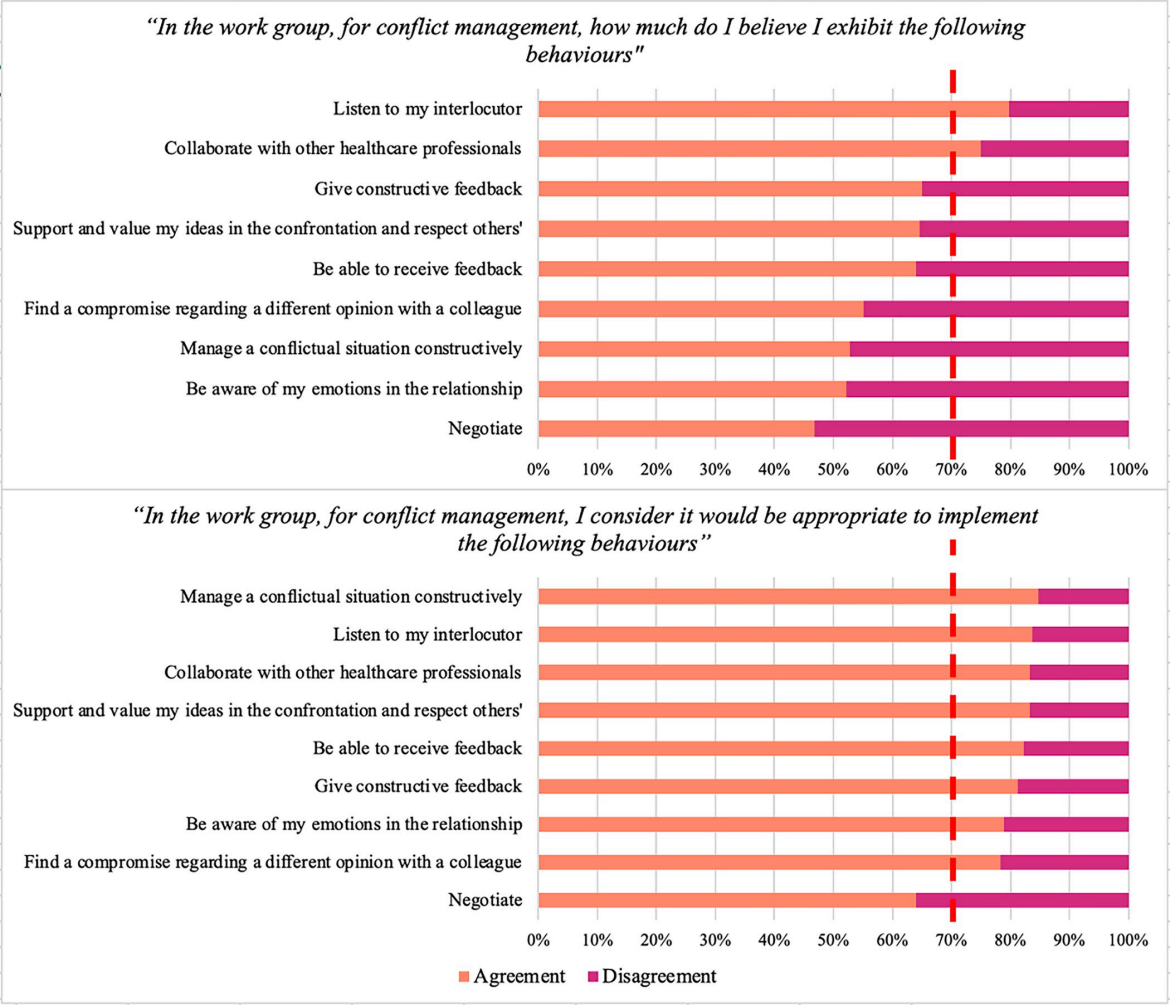
Perception of conflict management behaviours and appropriateness of these behaviours for effective conflict resolution. Note: the red lines represent 70% of the agreement.

In the context of conflict management within the workgroup, the analysis of statements related to the perception of engaging in certain behaviours related to conflict management revealed that agreement was found for only two out of ten statements (Fig 1). The exact percentage responses on the Likert scale are reported in Table 2. As far as the statements indicating which behaviours they believed would be appropriate, agreement was found in eight statements out of nine (Fig 1).

**Table 2:** Percentages of agreement/disagreement with the statements related to the perception of exhibiting certain behaviours related to conflict management and to which behaviours they believed would be appropriate for conflict management.

Behaviours	Fully Agree	Agree	Neither Agree Nor Disagree	Disagree	Fully Disagree	Fully Agree	Agree	Neither Agree Nor Disagree	Disagree	Fully Disagree
	*“In the work group*, *for conflict management*, *how much do I believe I exhibit the following behaviours”*					*“In the work group*, *for conflict management*, *I consider it would be appropriate to implement the following behaviours”*				
	N (%)	N (%)	N (%)	N (%)	N (%)	N (%)	N (%)	N (%)	N (%)	N (%)
Listen to my interlocutor	75 (37.0)	87 (42.9)	30 (14.8)	8 (3.9)	3 (1.4)	124 (61.1)	46 (22.7)	23 (11.3)	5 (2.5)	5 (2.5)
Be aware of my emotions in the relationship	39 (19.2)	67 (33.0)	68 (33.5)	23 (11.3)	6 (3.0)	108 (53.2)	52 (25.6)	28 (13.8)	9 (4.4)	6 (3.0)
Support and value my ideas in the confrontation and respect others’	54 (26.6)	77 (37.9)	48 (23.7)	20 (9.9)	4 (2.0)	106 (52.2)	63 (31.0)	22 (10.8)	7 (3.5)	5 (2.5)
Collaborate with other healthcare professionals	71 (35.0)	81 (39.9)	39 (19.2)	9 (4.4)	3 (1.5)	118 (58.1)	51 (25.1)	25 (12.3)	6 (3.0)	3 (1.5)
Negotiate	22 (10.8)	73 (36.0)	70 (34.5)	29 (14.3)	9 (4.4)	65 (32.0)	65 (32.0)	52 (25.6)	11 (5.4)	10 (4.9)
Give constructive feedback	50 (24.6)	82 (40.5)	44 (21.7)	24 (11.8)	3 (1.5)	104 (51.23)	61 (30.1)	24 (11.8)	11 (5.4)	3 (1.5)
Be able to receive feedback	46 (22.7)	84 (41.4)	44 (21.7)	22 (10.8)	7 (3.5)	105 (51.7)	62 (30.5)	24 (11.8)	8 (3.9)	4 (2.0)
Find a compromise regarding a different opinion with a colleague	28 (13.8)	84 (41.4)	64 (31.5)	24 (11.8)	3 (1.5)	79 (38.9)	80 (39.4)	33 (16.3)	6 (3.0)	5 (2.5)
Manage a conflictual situation constructively	39 (19.2)	68 (33.5)	63 (31.0)	27 (13.0)	6 (3.0)	122 (60.1)	50 (24.6)	21 (10.3)	7 (3.5)	3 (1.5)

N, number.

**Fig 2:**
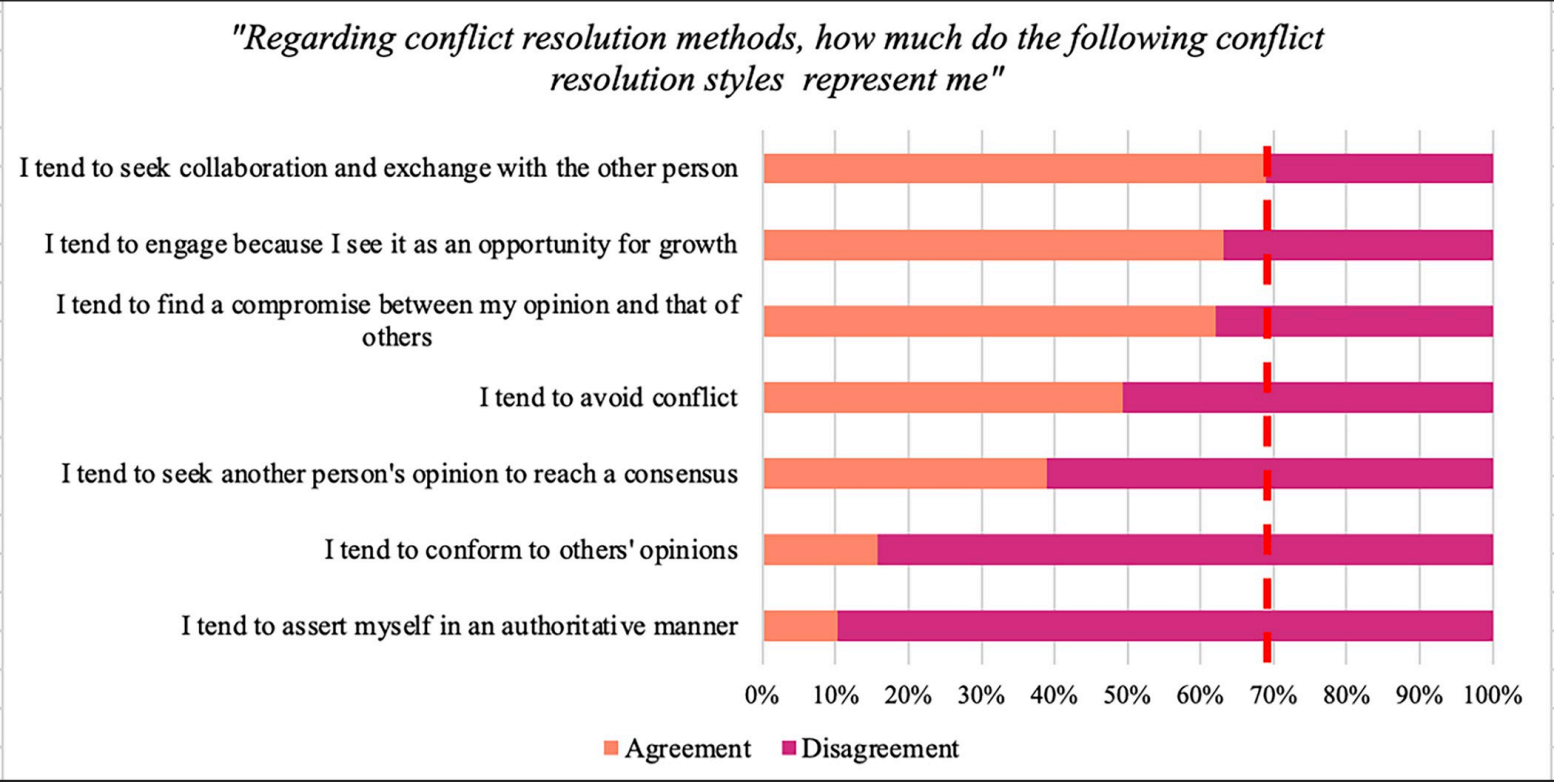
Adoption of conflict management styles. Note: the red line represents 70% of the agreement.

The exact percentage responses on the Likert scale are reported in Table 2. Finally, in the statements regarding the conflict management styles that best represented their actions, agreement was found for none of them (Fig 2). The exact percentage responses on the Likert scale are reported in Table 3.

**Table 3:** Percentages of agreement/disagreement with the statements related to the conflict resolution styles.

Statements	Fully Agree	Agree	Neither Agree Nor Disagree	Disagree	Fully Disagree
	N (%)	N (%)	N (%)	N (%)	N (%)
I tend to assert myself in an authoritative manner	4 (2.0)	17 (8.4)	53 (14.8)	82 (40.4)	47 (23.2)
I tend to conform to others’ opinions	5 (2.5)	27 (13.3)	56 (28.0)	72 (35.5)	43 (21.2)
I tend to avoid conflict	30 (14.8)	70 (34.5)	71 (35.0)	25 (12.3)	7 (3.5)
I tend to find a compromise between my opinion and that of others	25 (12.3)	101 (50.0)	51 (25.1)	18 (8.9)	8 (3.9)
I tend to seek another person’s opinion to reach a consensus	19 (9.4)	60 (29.6)	77 (38.0)	35 (17.2)	12 (5.9)
I tend to seek collaboration and exchange with the other person	49 (24.1)	90 (44.3)	42 (20.7)	12 (5.9)	9 (4.4)
I tend to engage because I see it as an opportunity for growth	55 (27.1)	73 (36.0)	49 (24.1)	18 (8.9)	8 (3.9)

N, number.

### Section 3: Training experiences and needs in conflict management

In the realm of conflict management skills and educational needs, agreement was found for the statement revolving around the importance of being trained in conflict management. Specifically, 85.7% indicated their agreement with the statement, “I consider it important for my profession to be trained in understanding and managing conflict situations within the workgroup”. Conversely, as regards the question, “Currently, I find my conflict resolution skills in the work context satisfactory”, 27.6% of the respondents agreed with this statement. When it came to the statements regarding coping with conflicts under different relational situations, no agreement was found in any statements regarding finding it difficult to deal with colleagues of the same profession, colleagues from another profession, colleagues who supervise them, and professional figures they manage. The exact percentage responses on the Likert scale are reported in Table 4.

**Table 4:** Percentages of agreement/disagreement with the statements related to the training experiences and needs in conflict management.

Statements	Fully Agree	Agree	Neither Agree Nor Disagree	Disagree	Fully Disagree
	N (%)	N (%)	N (%)	N (%)	N (%)
*Training Experiences in Conflict Management*					
Currently, I consider my skills in addressing conflicts in the work context to be satisfactory	7 (7.4)	41 (20.2)	66 (32.5)	67 (33.0)	14 (6.9)
I consider it important for my profession to be trained in understanding and managing conflict situations within the workgroup	106 (52.2)	68 (33.5)	23 (11.3)	4 (2.0)	2 (1.0)
*Training Needs in Conflict Management*					
Conflict with colleagues of the same profession	18 (8.9)	63 (31.0)	48 (23.7)	61 (30.1)	13 (6.4)
Conflict with colleagues from another profession	35 (17.2)	79 (38.9)	57 (28.1)	25 (12.3)	7 (3.5)
Conflict with colleagues who supervise me	49 (24.1)	61 (30.1)	49 (24.1)	33 (16.3)	11 (5.4)
Conflict with professional figures that I supervise	15 (7.4)	22 (10.8)	76 (37.4)	44 (21.7)	46 (22.7)

N, number.

### Section 4: Beliefs about factors influencing conflict management and conflict management impact

**Fig 3:**
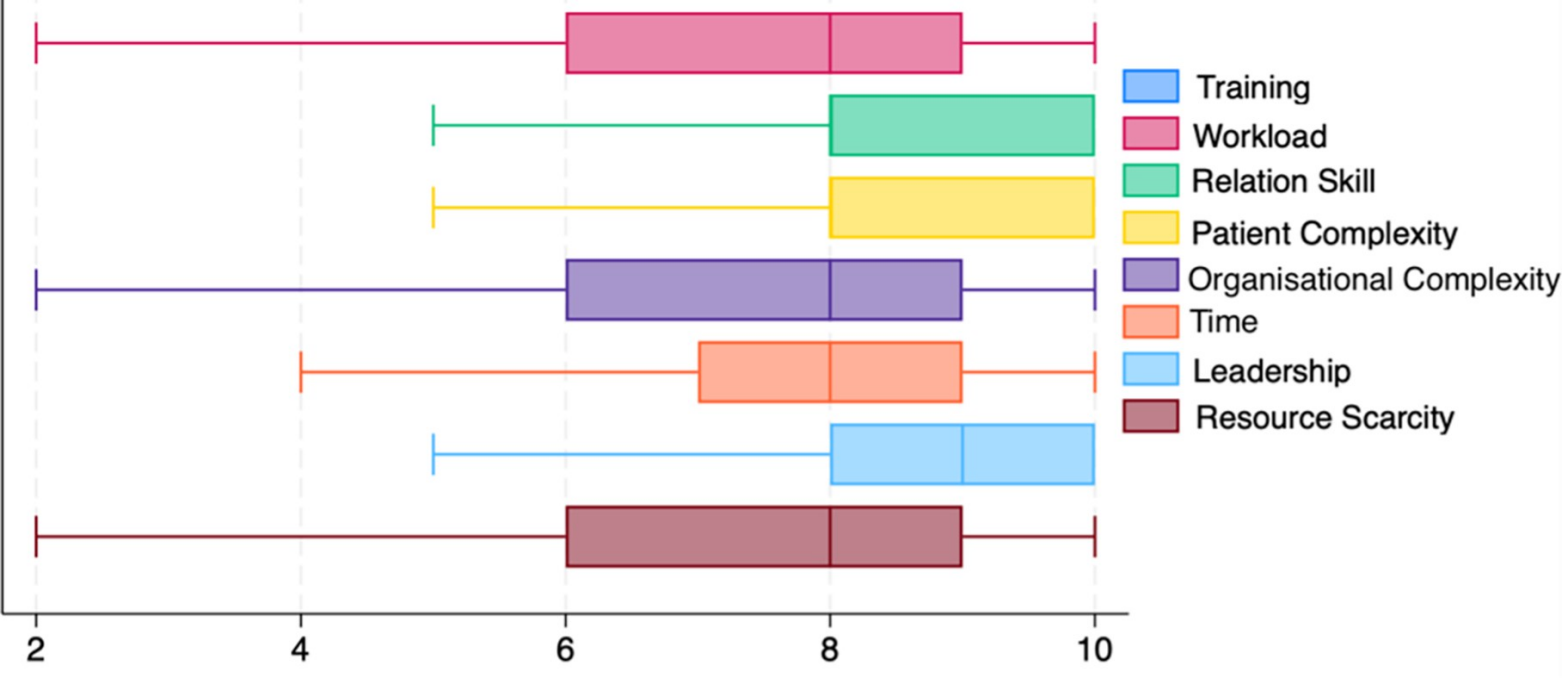
Levels of influence of various factors on conflicts.

In Fig 3, the medians and distribution of responses on factors that, according to the participants, can influence conflict management within the workgroup on a scale of 1–10 are reported. The medians (and the first and third quartiles) related to each factor are reported hereafter. All factors were considered to substantially impact conflict management (medians between eight and nine). Specifically, a median of nine was obtained with respect to workload (8, 0), relation skill (8,10), leadership (8,10), and resource scarcity (6, 9), while patient complexity (6, 9), organisational complexity (7, 9), and time received (6, 9) a median of eight.

Regarding how conflict influences various aspects of work, the following three factors were considered: quality of care, biopsychosocial approach, and well-being. All factors reached a median of nine. Specifically, the first and third quartiles were six and ten for quality of care and biopsychosocial approach and eight and ten for well-being.

## Discussions

This paper examined the perceived competencies, attitudes, and training needs of a cohort of Italian physiotherapists regarding conflict management in the workgroup. Regarding conflict frequency, more than half report the need of engaging in conflict management as “frequent” or “sometimes.” Overall, our results demonstrated that the surveyed physiotherapists were aware of the importance of conflict management strategies. Nevertheless, they did not feel well-trained and confident in dealing with intra- and inter-professional conflicts. In terms of exhibited specific conflict management behaviours, there was high agreement on "listening to the interlocutor" and "collaborating with other healthcare professionals." Additionally, more than 60% of the participants reported exhibiting behaviours such as "give constructive/be able to receive feedback" and "support and value my ideas in the confrontation and respect others". Interestingly, there was a perception of less use and perceived appropriateness of negotiation in conflict management. The recognition of "awareness of emotions in the relationship" appears to be perceived as less implemented. Given the significance of being attuned to emotions as a crucial element of emotional intelligence, which in turn plays a pivotal role in fostering effective relational skills, this aspect warrants attention in future training endeavours [24]. Although participants recognised that these behaviours would be appropriate for conflict management, there was a discrepancy between perceived use and the theoretical appropriateness, suggesting a possible difficulty in applying theoretical knowledge in practice.

While no agreement was found concerning conflict management styles, discernible trends in adopting certain styles were observed, notably a greater inclination towards seeking compromise, and collaboration. However, nearly half of the participants also tend to adopt avoidance behaviours, with only a small percentage leaning towards passivity or aggressiveness in managing conflicts. Comparable results were identified in studies involving other healthcare professional groups, such as professional nurses, where a preference for constructive/positive conflict management styles (e.g., integration, collaboration) outweighed destructive styles [25]. Remarkably, more than half of the participants in our study demonstrated a predisposition to view conflict management as an opportunity for growth, indicating a positive perspective on conflict as a catalyst for team development. This aligns with discussions highlighting the transformative potential of conflict, emphasising that effective handling can lead to improvements, such as increased efficiency and collective learning for the entire team [26, 27].

If just over one-third of participants found it challenging to collaborate with physiotherapy colleagues, more than half of the participants reported difficulties in managing conflicts with other professionals. Correspondingly, Perreault et al. noted higher satisfaction in professional interactions, specifically with other physiotherapists [11]. Additionally, our study did not delve into potential variations in interactions with different professionals, as observed by Perreault et al., where interactions with medical specialists were less satisfying than those with family physicians. Examining the relationship status, managing conflicts with individuals in coordinating roles appears more challenging for the participants. Power and status differences play a significant role in conflict dynamics [28]. In a study by Bajwa et al., 58% of conflicts occurred between professionals at different hierarchical levels, notably between individuals with lower status (e.g., residents) and those with higher status (e.g., attending physicians) [29]. This status dynamic often dissuaded those with lower status from addressing their concerns with superiors. Recognising these distinctions in relationships can be instrumental in shaping the design of training programs.

Examining training needs related to conflict management in workgroup, it was noteworthy that only less than one third of participants were content with their current conflict management skills. Correspondingly, nearly all desired training in this area, aligning with findings in studies on healthcare professionals like for example nurses [30]. While studies on private-sector physiotherapists, such as Perreault et al., suggested satisfaction with training, our study underscores dissatisfaction with conflict management skills [11].

Delving into beliefs about factors influencing conflict management, the data suggested attributing conflicts to both personal factors (relationship skills) and organisational and external factors (overload, leadership, lack of resources and time, organisational factors). Workload emerged as a key obstacle, echoing Perreault et al.’s findings [11]. These results might indicate participants’ awareness of the intricate factors affecting conflict management in the healthcare context [5]. Finally, participants demonstrated an understanding of the potential negative consequences of inadequate conflict management, acknowledging its impact on care quality, integration work (adoption of a bio-psycho-social model), and practitioner well-being, impacts which have been highlighted in the literature.

The study presented several limitations. Limitations include an uncalculated response rate, risking sampling bias. Furthermore, the research lacked a targeted examination of specific work settings, potentially neglecting challenges unique to particular environments. Factors tied to the intricacies of the work environment could render certain contexts especially demanding, leading to heightened stress levels and complicating conflict management [27, 31, 32]. Future research should target physiotherapists in different rehabilitation settings. Furthermore, relying on self-reported measures posed limitations, as perceptions may not align with practical implementation and lead to recall bias. Role-playing or real-life observations in real context could offer a more accurate assessment of level or relational skills and educational needs, as previously suggested in the context of communication skills training for healthcare professionals [33]. Given the exploratory nature of this study, closed questions on a Likert scale were used, limiting the depth of insights. Finally, we could not compute the sample size as there are no data available on the number of Italian physiotherapists working in team.

## Conclusions

In conclusion, this study offered a unique perspective on the perceived competencies, attitudes, and training needs of Italian physiotherapists regarding conflict management in workgroup. Participants generally perceived to exhibit a positive attitude and constructive approaches to conflict yet expressed dissatisfaction with their conflict management skills, emphasising the importance of dedicated training. These findings underscored the necessity of implementing specific training programs, potentially through effective modalities like role-playing or simulations, known to enhance interprofessional collaborative practice and patient well-being. According to participants, despite the acknowledged significance of conflict management training, both personal and organisational factors appear to have significant impact. This study serves as an initial step towards guiding physiotherapists’ education in conflict management and, more broadly, providing insights for healthcare providers to target areas of improvement for optimal interprofessional collaboration. Our findings underscored the potential benefits of incorporating conflict management training into the professional development of physiotherapists. The past observed correlation between effective conflict management strategies and improved workplace well-being and enhanced care quality suggests that addressing conflict resolution skills may contribute significantly to the overall effectiveness of physiotherapy practice.

## Supporting information

S1 File Survey instrument. Survey instrument translated in English from Italian language.
(PDF)
