# Supplementary material for: Perceived competences, attitudes, and training needs in conflict management among a cohort of Italian physiotherapists: A cross-sectional survey study
Source: PLoS One. 2024 Jul 26;19(7):e0306095. doi: 10.1371/journal.pone.0306095 (PMC11280247; doi:10.1371/journal.pone.0306095)
Supplement: S1 File — Survey instrument translated in English from Italian language. (PDF) [file pone.0306095.s001.pdf]

UNIVERSITÀ DEGLI STUDI DI GENOVA

Dipartimento di Neuroscienze, Riabilitazione, Oftalmologia, Genetica e Scienze Materno-Infantili

SECTION 1 – Socio-Demographic, Professional Data and Conflict Frequency

1. Professional profile of affiliation:
    - Physiotherapist
    - Other (end of the questionnaire)

2. In the course of your professional activity, do you find yourself working in teams / work groups?
    - Yes, always
    - Yes, sometimes
    - No, never (end of the questionnaire)

3. Age: ___

4. Gender identification: Male / Female / Other _____ / I prefer not to answer

5. For how many years have you been working as a Physiotherapist/TeRP?
    - Less than one year
    - 1 to 5 years
    - 6 to 10 years
    - More than 10 years

6. With which professional figures do you mainly work? (Multiple answers possible)
    - Nurse
    - Healthcare Assistant
    - Physician
    - Speech Therapist
    - Professional Educator
    - Psychologist
    - Occupational Therapist
    - Psychiatric Rehabilitation Technician
    - Physiotherapist
    - Other______________________

7. In terms of frequency, how often do you find yourself in situations requiring the management of a potential conflict in your work group?
    - Never
    - Rarely (Less than once a month)
    - Occasionally (Once a month)
    - Often (More than once a month)
    - Daily

UNIVERSITÀ DEGLI STUDI DI GENOVA

Dipartimento di Neuroscienze, Riabilitazione, Oftalmologia, Genetica e Scienze Materno-Infantili

In the questionnaire, the term "conflict" refers to interpersonal and/or group conflicts within the work context. By conflict, we mean a situation of incompatibility, disagreement, or discord that arises when there are contrasting positions on ideas, resources, skills, needs, or when the goals of one party interfere with the goals of another.

SECTION 2 - Attitudes Toward Conflict and Perceived Competences in Conflict Management

**8. In the work group, for conflict management, how much do I believe I exhibit the following behaviours:**					
	**Fully Agree**	**Agree**	**Neither Agree Nor Disagree**	**Disagree**	**Fully Disagree**
Listen to my interlocutor					
Be aware of my emotions in the relationship					
Support and value my ideas in the confrontation and respect others'					
Collaborate with other healthcare professionals					
Negotiate					
Give constructive feedback					
Be able to receive feedback					
Find a compromise regarding a different opinion with a colleague					
Manage a conflictual situation constructively					
**9. In the work group, for conflict management, how important do I consider the following behaviours:**					
Listen to my interlocutor					
Be aware of my emotions in the relationship					

Support and value my ideas in the confrontation and respect others'					
Collaborate with other healthcare professionals					
Negotiate					
Give constructive feedback					
Be able to receive feedback					
Find a compromise regarding a different opinion with a colleague					
Manage a conflictual situation constructively					
**10. Regarding conflict resolution methods, how much do the following conflict resolution methods represent me:**					
	**Fully Agree**	**Agree**	**Neither Agree Nor Disagree**	**Disagree**	**Fully Disagree**
I tend to assert myself in an authoritative manner					
I tend to conform to others' opinions					
I tend to avoid conflict					
I tend to find a compromise between my opinion and that of others					
I tend to seek another person's opinion to reach a consensus					

I tend to seek collaboration and exchange with the other person					
I tend to engage because I see it as an opportunity for growth					

SECTION 3: Training Experiences and Needs in Conflict Management

11. Currently, I consider my skills in addressing conflicts in the work context to be satisfactory:

Fully Agree	Agree	Neither Agree Nor Disagree	Disagree	Fully Disagree

12. I consider it important for my profession to be trained in understanding and managing conflict situations within the work group:

Fully Agree	Agree	Neither Agree Nor Disagree	Disagree	Fully Disagree

**13. Within the work group, I find the following situations challenging to manage:**					
	**Fully Agree**	**Agree**	**Neither Agree Nor Disagree**	**Disagree**	**Fully Disagree**
Conflict with colleagues of the same profession					
Conflict with colleagues from another profession					
Conflict with colleagues who supervise me					
Conflict with professional figures that I supervise					

UNIVERSITÀ DEGLI STUDI DI GENOVA
Dipartimento di Neuroscienze, Riabilitazione, Oftalmologia, Genetica e Scienze Materno-Infantili

SECTION 4: Beliefs About Factors Influencing Conflict Management and Conflict Management Impact

**14. How much do I believe the following factors can influence conflict management within the work group, on a scale from 0 to 100?**	
Time dedicated to professional discussion	**0-100**
Perceived workload	**0-100**
Personal relational skills	**0-100**
Complexity of the clientele	**0-100**
Organizational variables	**0-100**
Leadership	**0-100**
Scarcity of resources	**0-100**

**15. In terms of impact, in your opinion, how much does conflict management within the work group influence the following areas on a scale from 0 to 100?**	
The quality of care provided	**0-100**
The use of a bio-psychosocial approach with the patient	**0-100**
The atmosphere in your work environment	**0-100**
Your well-being	**0-100**
